# Die Kunst der Gipskeilung im Kindes- und Jugendlichenalter

**DOI:** 10.1007/s00064-025-00897-7

**Published:** 2025-05-06

**Authors:** Daniel Frühwirt, Kai Ziebarth

**Affiliations:** 1 Abteilung für Orthopädie und Unfallchirurgie, Universitätsklinikum St. Pölten, Dunantplatz 1, 3100 St. Pölten, Österreich; 2 Abteilung Kinderorthopädie, Kinderchirurgische Universitätsklinik, Inselspital Bern, Freiburgstr., 3010 Bern, Schweiz

**Keywords:** Fraktur, Vorderarm, Keilen, Unterschenkel, Stellungskorrektur, Forearm, Bone fractures, Wedging, Tibia fracture, Closed fracture reduction

## Abstract

**Operationsziel:**

Stellungskorrektur mittels Gipskeilung, wodurch es zu einem Redressement und einer Retention des Knochenbruches in lokalisations- und altersabhängig tolerabler Frakturstellung kommt. Effiziente und zumutbare Behandlung ohne Notwendigkeit einer operativen Intervention.

**Indikationen:**

Unterarmfrakturen distales Schaftdrittel sowie Frakturen der distalen Radiusmetaphyse. Unterschenkel- bzw. Tibiaschaftfrakturen ab Midschaft sowie Frakturen der distalen Tibiametaphyse.

**Kontraindikationen:**

Proximale und mittlere Vorderarmfrakturen. Vollständige Dislokation ohne Knochenkontakt der Frakturenden. Gelenkfrakturen (ausschließlich epiphysäre Verletzungen). Sehr junge Patienten, weil eine konklusive Rückmeldung des Kindes bezüglich Schmerz/Neurologie häufig sehr schwierig ist. Offene Frakturen.

**Operationstechnik:**

Anlage eines zum Keilen vorbereiteten Gipsverbandes, am 8. bis 10. Tag nach Trauma Stellungskorrektur durch aufklappende Gipskeilung, die in der maximalen Konkavität der Fraktur zur Wirkung kommen soll.

**Weiterbehandlung:**

Wöchentliche Kontrollen der Verbandsanordnung bis zur Gipsabnahme, vorzeitige selbstständige Wiedervorstellung bei Schmerzen (Aufklärung!). Fixationsdauer beim Kind 4 Wochen, bei Adoleszenten 4 bis 6 Wochen.

**Ergebnisse:**

Im Rahmen einer Studie der Autoren mit einem Beobachtungszeitraum von 8 Jahren wurden in der Klinik in Sankt Pölten 199 Frakturen bei Kindern nachkontrolliert. In lediglich 2 Fällen frustrane Keilung mit operativem Verfahrenswechsel (proximaler Radius – ESIN [elastische stabile intramedulläre Nagelosteosynthese], distale Tibiametaphyse – K[Kirschner]-Drähte), weiter Refraktur nach Gipsabnahme bei 4 von insgesamt 78 gekeilten Grünholzfrakturen an der Speiche (Refrakturrate von 5 %, weit unter den üblichen Literaturangaben). Bei 96 % des Patientenguts konnte das Therapieziel durch die Gipskeilung erreicht werden.

## Abstract (en)

**Objective:**

Correction of pediatric fractures by cast wedging to achieve acceptable positioning for conservative fracture management. Efficient and convenient treatment to avoid invasive manipulation or hospitalization.

**Indications:**

Fractures of distal forearm shaft or distal metaphyseal forearm. Tibial shaft fractures from midshaft to distal metaphyseal region.

**Contraindications:**

Proximal and middle forearm fractures. Complete dislocation. Articular fractures. Very young children (compliance problems). Open fractures.

**Surgical technique:**

Immediate cast application for fracture treatment as usual. After 8–10 days wedging of the cast at concavity of fracture site leads to gentle fracture reduction within a few days.

**Postoperative management:**

Depending on the age of the patient and location of the fracture, weekly visits to look for any discomfort or pain while cast treatment after wedging. Depending on the age of patient, duration of the cast is 4–6 weeks.

**Results:**

A recent analysis of 199 fractures in Sankt Pölten (average age 8.9 years) showed low refracture rates. In only 2 cases did unsuccessful wedging lead to surgical treatment (proximal radius—elastic stable intramedullary nailing [ESIN], distal tibial metaphysis—K-wires). Furthermore, refracture after cast removal occurred in 4 out of a total of 78 greenstick fractures of the radius (refracture rate 5%, well below the usual figures reported in the literature). The treatment goal was achieved with cast wedging in 96% of the patient population.

## Vorbemerkungen

Die Gipskeilung ist eine konservative Behandlungsmethode von ausgewählten Frakturen am Unterarm bzw. am Unterschenkel, die erstmals 1936 in der Literatur erwähnt wurde [6]. Sie hat damals vorwiegend bei den Erwachsenen Anwendung gefunden und ist im Laufe der Zeit durch die entstandenen Osteosyntheseverfahren wieder in Vergessenheit geraten. In der Kindertraumatologie ist die Gipskeilung ein ambulantes Verfahren in der Behandlung von Knochenbrüchen am distalen Unterarm sowie am Unterschenkel im Schaftbereich und an der distalen Metaphyse, die gesamt rund 35 % aller Frakturen am wachsenden Skelettsystem ausmachen [11]. Im Vergleich zu alternativen Therapiemethoden (Repositionen mit oder ohne Anästhesieverfahren, Osteosynthesen) liegt der Vorteil in der Effizienz der Behandlung und der Minimierung von iatrogenen Komplikationsmöglichkeiten. Da es sich lediglich um ein Redressement der Fraktur bei bereits vorhandenem Fixationskallus handelt, kommt es, zumindest bei achsengerechter Konsolidierung, auch zu einer zeitlichen Limitierung der obligatorisch auftretenden stimulativen Wachstumsstörung. Dies ist v. a. bei Frakturen an der unteren Extremität von Relevanz. In unserer Klinik wurde 2016 mit der Gipskeilung begonnen, mittlerweile ist sie eine etablierte Behandlungsmethode, die einen fixen Stellenwert in der Frakturbehandlung von Kindern und Jugendlichen hat. Als Material verwenden wir Weißgips, in anderen Häusern wird auch mit Scotchcast gekeilt, Soft-Cast-Verbände sind ungeeignet, weil zu weich. Die individuelle Auswahl der Materialien wie z. B. zur Gipspolsterung (Filz/Watte/Kunststoff) oder zum Keilen (Kork/Holz) obliegt den lokalen Gegebenheiten der jeweiligen Klinik.

Da es in der Literatur an wirklich hilfreichen Arbeiten über diese konservative Therapiemöglichkeit mangelt, soll dieser Artikel dazu beitragen, dem Leser die Gelegenheit zu geben, die immer mehr in Vergessenheit geratene Therapie der Gipskeilung in die Praxis umsetzen zu können. Bezüglich historischer Entwicklung [2, 6] und biomechanischer Überlegungen [1, 4, 10] wird von den Autoren auf die Literaturangaben verwiesen.

## Operationsprinzip und -ziel

Behandlungsprinzip ist die Stellungskorrektur einer primär oder sekundär dislozierten Fraktur am Unterarm bzw. Unterschenkel im Wachstumsalter durch aufklappende Gipskeilung, wodurch es zu einem Redressement und einer Retention des Knochenbruches in lokalisations- und altersabhängig tolerabler Frakturstellung kommt.

Hierzu wird bei geeigneten Knochenbrüchen am Unfalltag ein Spaltgipsverband angelegt, der nach rund 4 Tagen auf einen zur Keilung vorbereiteten Gips gewechselt wird. Am 8. bis 10. Tag erfolgt eine Röntgenkontrolle mit, sofern erforderlich, nachfolgender Gipskeilung. Das Keilungsergebnis wird radiologisch dokumentiert. Im Regelfall Belassen der gekeilten Gipsanordnung für weitere 3 Wochen, in Ausnahmefällen, wie bei komplexen Tibiaschaftfrakturen im Adoleszentenalter, kann die Gipsbefristung insgesamt auf maximal 6 Wochen ausgedehnt werden. Nach Gipsabnahme Durchführung eines Konsolidierungsröntgens.

Behandlungsziel am *distalen Unterarm* ist eine Konsolidierung der Fraktur in ggf. tolerabler Restachsenabweichung, die aufgrund der benachbarten hochpotenten und langlebigen Fuge im Laufe des weiteren Wachstums altersabhängig zuverlässig korrigiert wird [7]. Das Ausheilungsergebnis muss jedoch für den Patienten zumutbar sein, äußerlich sichtbare Fehlstellungen, wenn auch durch das Remodelling nur passager, sollten keine verbleiben.

Unser Behandlungsziel am *Unterschenkel *ist zur Vermeidung von relevanten Beinlängendifferenzen und/oder bleibenden Fehlstellungen altersunabhängig die achsengerechte Knochenbruchheilung. Die in der gängigen Literatur [11] angegebenen tolerablen Restachsenabweichungen sollten unserer Ansicht nach, wenn möglich, am Unterschenkel vermieden werden.

*Tolerable Restdislokation am Unterarm* [11]:

- < 12. Lebensjahr (LJ.):
  - Sagittalebene: 30/40°
  - Frontalebene: 10/20°
- > 12. LJ.:
  - Beide Ebenen: 0–10/20° (abhängig vom Reifegrad der Fugen)

*Tolerable Restdislokation Unterschenkel* [11]:

- Altersunabhängig:
  - Varus 5°/Valgus 0°
  - Re- und Antekurvation bis 10° (abhängig vom Reifegrad der Fugen)

## Vorteile

- Keine Hospitalisation
- Wegfall von Narkose- und Operationsrisiko, keine Provokation von zusätzlichen iatrogenen Wachstumsstörungen
- Stressfreie Behandlung für den Patienten (keine Blutabnahmen, Verbandswechsel, Nahtentfernung, Reoperation etc.)
- Kostengünstiges effizientes Verfahren

## Nachteile

- Längere Immobilisierung durch Gipsverband im Vergleich zu operativen Techniken mit funktioneller Nachbehandlung (ESIN [elastische stabile intramedulläre Nagelosteosynthese], Fixateur externe)
- Logistische Herausforderung, genaue Einhaltung des Zeitplans für die einzelnen Behandlungsschritte notwendig
- Geeignete Infrastruktur zur konservativen Frakturbehandlung, geschultes Gipspersonal und persönliches Engagement erforderlich
- Längere Lernkurve aufgrund kaum vorhandener wissenschaftlicher Publikationen und/oder fehlender/verloren gegangener Expertise in vielen Hospitälern

## Indikationen

Grundvoraussetzung für die Indikationsstellung sowohl am Unterarm als auch am Unterschenkel ist ein bestehender Knochenkontakt der Frakturfragmente, das Ausmaß der Achsenabweichung wird limitiert durch die Zumutbarkeit für den Patienten (Schmerzen, gefährdete Weichteile). Die periphere Durchblutung, Motorik und Sensibilität sind immer zu überprüfen und müssen erhalten sein.

- *Unterarm:*
  - Distale metaphysäre Radiusfraktur (± Beteiligung der Ulna)
  - Radiusfraktur distales Schaftdrittel
  - Unterarmfraktur distales Schaftdrittel mit Achsenabweichung beider Knochen in gleicher Ebene
- *Unterschenkel:*
  - Tibiaschaftfraktur (± Grünholzkomponente Fibula)
  - Distale metaphysäre Tibiafraktur (± Grünholzkomponente Fibula)

## Kontraindikationen

- Vollständig dislozierte, verkürzte Frakturen
- Unterarmfrakturen im distalen Schaftdrittel mit Achsenabweichung der Knochen in verschiedenen Ebenen
- Unterarmfrakturen im proximalen und mittleren Schaftdrittel
- Kinder < 3 Jahre (unzureichendes Feedback des Patienten während und nach der Keilung)
- Offene Frakturen

## Patientenaufklärung

- Allgemeine Prinzipien der Gipskeilung
- Zeitlicher Ablauf
- Behandlungsalternativen bei Nichterreichen des Therapieziels
- Spontankorrektur von tolerabler Restachsenabweichung am distalen Unterarm bei Patienten unter 10 Jahren
- Refrakturgefahr bei Grünholzfrakturen am distalen Unterarm
- Genaue Erklärung der einzelnen Schritte unmittelbar vor der Keilung

## Operationsvorbereitungen

- Analyse der primären Röntgenaufnahme und der Stellungskontrolle vor der Keilung
- Bestimmung der Höhe und der Richtung der Keilungsstelle

## Instrumentarium

**Abb. 1:**
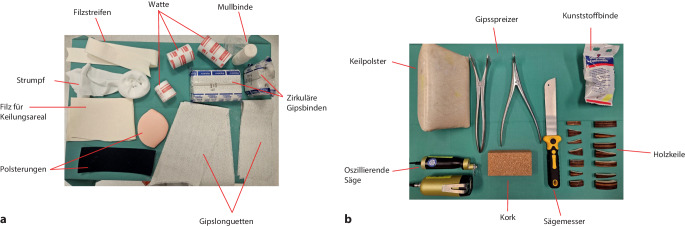
**a**) Standartmaterialen für die Anlage eines zur Keilung vorbereiteten Gipsverbandes: Gipslonguetten und zirkuläre Gipsbinden in verschiedenen Dimensionen, Filzstreifen zum Schutz der Haut bei der Gipsabnahme, elastischer Strumpf, Mullbinde zur Fixierung der Gipslonguette am Unterarm mittels Hohlhandtour, Watte zur Polsterung an den Gipsenden, zirkulärer Filz auf Höhe des geplanten Keilungsareal, Polsterungsmaterialien zur Vermeidung von Druckstellen; **b**) Standartmaterialien für die Gipskeilung: Oszilierende Säge, Keilpolster als Hypomochlion bei der Keilung am Unterarm, Holzkeile in verschiedenen Ausfertigungen und Dimensionen, ggf. Kork zum randständigen Ausfüllen des Keilungsspaltes, Sägemesser zum Zuschneiden des Korks, Kunststoffbinde zum definitiven Verschluss der Keilungsstelle

- *Anlage des zum Keilen vorbereiteten Gipsverbandes* (Abb. 1a): Gipslonguetten, zirkuläre Gipsbinden, elastischer Strumpf, längs eingelegter Filzstreifen (Hautschutz bei der Gipsabnahme mit der oszillierenden Säge), Filz (Hautschutz an der voraussichtlichen Keilungsstelle), Klebefilz (Polsterung an prädisponierten Lokalisationen zur Verhinderung von Druckstellen), Fersenpolster, Watte, Mullbinde für Hohlhandtour
- *Gipskeilung* (Abb. 1b): oszillierende Säge, Gipsspreizer, zugeschnittene Keile (Holz, Kork), Kunststoffbinde zum Verschluss der Keilungsstelle, ggf. Keilpolster mit hartem Kern als Hypomochlion am Unterarm

## Anästhesie und Lagerung

- Kein Anästhesieverfahren erforderlich und erwünscht, Feedback des Patienten bei der Keilung vonnöten
- Im Vorfeld der Gipsanlage und Keilung zeitgerechte häusliche orale Schmerzmittelgabe
- An der oberen Extremität erfolgt die Keilung im Sitzen, alternativ Durchführung in Rückenlage
- An der unteren Extremität Rückenlagerung

## Operationstechnik

### Technik am Unterschenkel

**Abb. 2:**
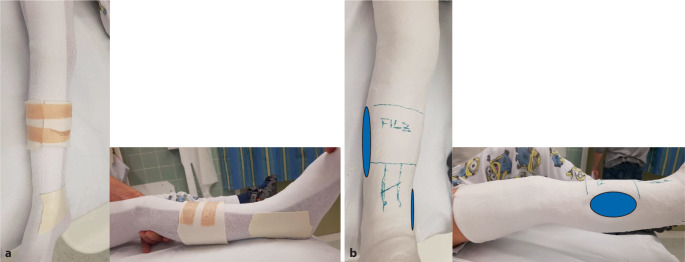
*Gipsanlage am Unterschenkel.* Um eine ausreichende Hebelwirkung zu erzielen, ist für die Gipskeilung am Unterschenkel ein Oberschenkelgips mit Streckstellung im Kniegelenk erforderlich. Ventralseitiges Einlegen eines dehnbaren Filzstreifens, faltenfreies Anlegen des Strumpfes. Anbringen des Klebefilzes an prädisponierten druckgefährdeten Stellen, standardmäßig Innen- und Außenknöchelregion und ggf. Tuberositas tibiae. Zum Schutz vor Druckstellen bei Korrektur einer Retrokurvationsfehlstellung zusätzlich Polsterung auch am Fußrücken und im Bereich der Ferse. Zirkuläres Positionieren eines rund 12 cm breiten Filzstreifens an der voraussichtlichen Keilungsstelle (**a**). Applikation von Watte am proximalen und distalen Gipsende. Als nächster Schritt wird eine zirkuläre Gipsbinde dünnschichtig aufgetragen. Dann Anlegen der bis zum proximalen Gipsende reichenden dorsalen Longuette, zusätzliche Verstärkung durch steigbügelartig aufgelegte zweite Gipslonguette, die bis zum Kniegelenk reicht. Vervollständigung durch zirkuläre Gipsrollen, wobei der Strumpf an den beiden Gipsenden vor der letzten Gipsbinde umgeschlagen und durch diese mitgefasst wird. Wichtig ist ein gefühlvolles Anmodellieren des Gipsverbandes entgegen der bestehenden oder zu erwartenden Fehlstellung, meist Varus bei primär unverschobenen isolierten Tibiaschaftfrakturen (**b**). Zur Vermeidung von unnötigen, die Keilung erschwerenden Zwischenräumen sollte der Gips bündig angelegt und bis zur Aushärtung permanent in Position gehalten werden. Zuletzt erfolgt zur Eingrenzung des möglichen Keilungsareals die Einzeichnung der Höhe des eingelegten zirkulären Filzstreifens. Idealerweise sind für Gipsanlage an der unteren Extremität ein Arzt, ein Gipsassistent und eine zusätzliche Hilfsperson zur Stabilisierung des Kniegelenks vonnöten

**Abb. 3:**
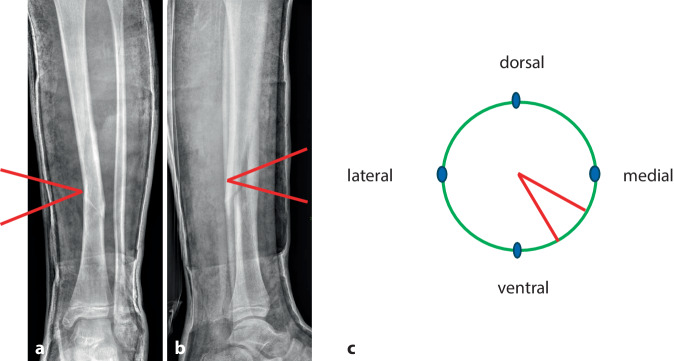
*Durchführung der Gipskeilung am Unterschenkel.* Knabe, 11 Jahre, Sturz beim Skifahren. Tibiaspiralfraktur Übergang mittleres in distales Schaftdrittel mit Grünholzkomponente an der Fibula. Am 8. Tag nach Trauma erfolgt eine radiologische Stellungskontrolle, bei der sich eine Dislokation in den Varus und eine Retrokurvationsstellung zeigen. Zunächst Analyse der Keilungsrichtung, die Wirkung des Keils muss in der maximalen Konkavität der Frakturfehlstellung(en) zum Tragen kommen. Zur Korrektur eines isolierten Varus erfolgt die Keilung von medial (**a**), die Beseitigung einer alleinigen Retrokurvation gelingt durch Keilung von ventral (**b**). Deshalb wird im angeführten Fall die Stellungskorrektur durch Aufdehnen des Gipsverbandes von ventromedial geplant (**c**)

**Abb. 4:**
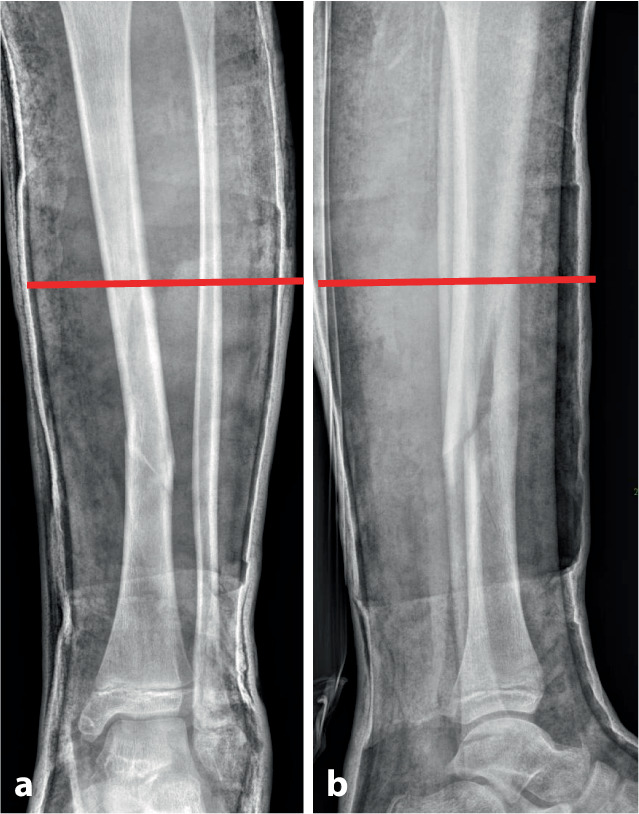
Als nächster Schritt wird die Höhe des Keils bestimmt, die bei diaphysären Quer- und kurzen Schrägfrakturen direkt über dem Bruch und bei längeren Spiralfrakturen am proximalen Bruchende liegt. Im distal metaphysären Bereich Positionierung des Keils rund eine Handbreite proximal der Fraktur. Da die genaue Lokalisation mitentscheidend für das Gelingen des Keilens ist, sollte, zumindest bei anspruchsvollen Keilungen (oder beim Ungeübten), die Bestimmung der Keilungshöhe unter Bildwandlerkontrolle erfolgen. **a**, **b** Keilungshöhe am proximalen Frakturende

**Abb. 5:**
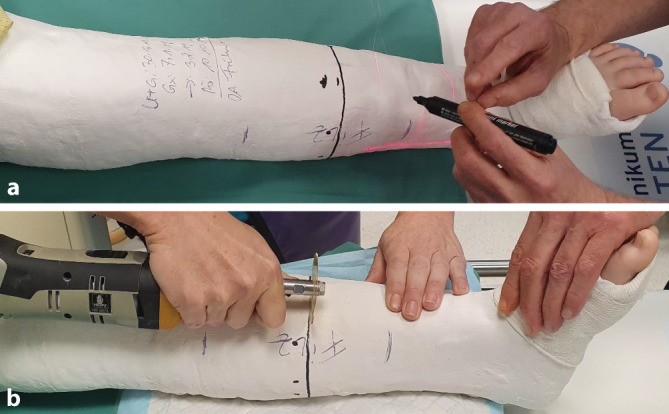
Es erfolgt jetzt das Einzeichnen der definitiven Keilungshöhe, wobei die Markierung über die halbe Zirkumferenz des Gipsverbandes reichen muss, um ein dosiertes Aufdehnen zu ermöglichen. Zur Vermeidung von Hautschäden ist unbedingt darauf zu achten, innerhalb des eingezeichneten Areals zu bleiben (**a**). Dann wird der Gips mit der oszillierenden Säge entlang der Linie eingeschnitten, wobei alle Schichten des Verbandes gespaltet werden müssen (**b**)

**Abb. 6:**
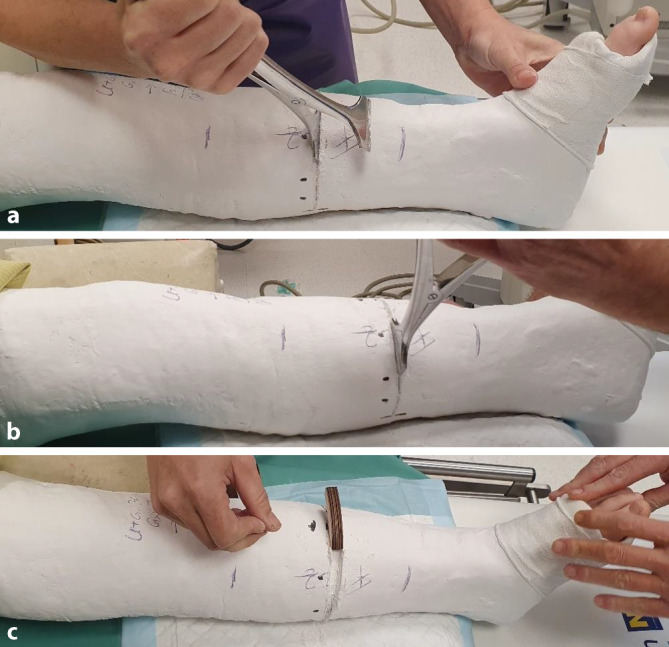
Zunächst minimales Aufspreizen des Gipses, dies sollte im zentralen Bereich der Markierung erfolgen, um ein Maximum an Hebelwirkung in der Konkavität der Fraktur zu erzielen (**a**, **b**). Passageres Einlegen eines Platzhalters und etwas Zuwarten (**c**). Wichtig ist die Kommunikation mit dem Patienten, bis auf geringe Beschwerden im Frakturbereich am Ende des Keilungsvorganges muss die Behandlung im Wesentlichen schmerzfrei sein

**Abb. 7:**
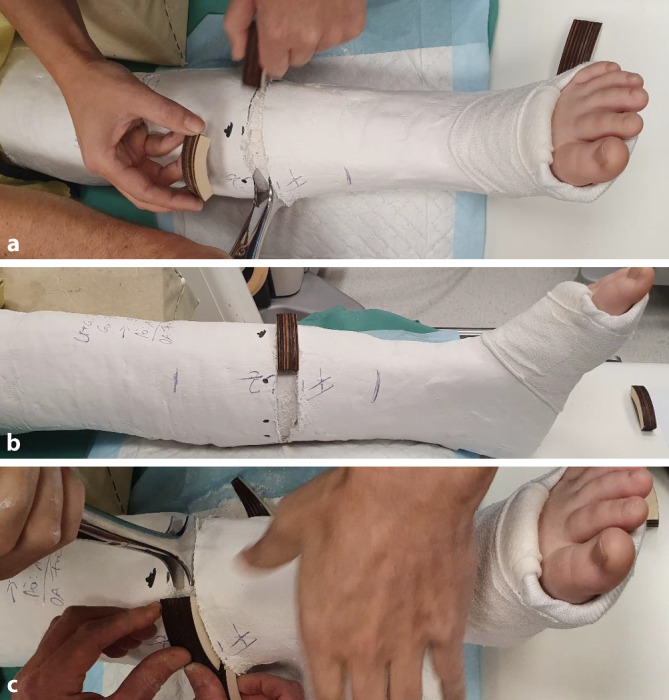
Schrittweise dosiertes weiteres Aufspreizen, wobei zum Offenhalten des vergrößerten Spaltes bis zum Einlegen des neuen Keils ein zweiter Spreizer zum Einsatz kommt. Ein „leichtes Ziehen“ in Frakturhöhe signalisiert meist die richtige Größe des Keils. **a** Dosiertes weiteres Aufspreizen, **b** größerer Keil als passagerer Platzhalter, **c** Positionierung des definitiven Keils

(Abb. 2, 3, 4, 5, 6, 7, 8 und 9).

**Abb. 8:**
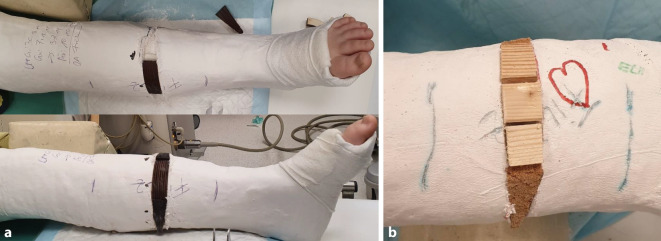
Stellungskontrolle mittels Bildwandler (achsengerechte Konsolidierung am Unterschenkel als Behandlungsziel). Ausfüllen des gesamten Spaltes mit hölzernen Platzhaltern, die in der Klinik des Autors in verschiedensten Ausfertigungen vorliegen. Zur Vermeidung eines „Fensterödems“ ist darauf zu achten, dass die Keilungsstelle zur Gänze mit den Platzhaltern ausgefüllt ist. Diese müssen in der richtigen Tiefe platziert sein, ein Druck auf den Filz ist zwecks Schonung der Weichteile strickt zu vermeiden (**a**). Alternativ kann der Spalt im Zentrum mit Holzstücken und am Rand mit entsprechend zugeschnittenen Korkstücken ausgefüllt werden (**b**). In der Literatur kommen auch vorgefertigte Platzhalter aus Hartplastik zur Anwendung [4]

**Abb. 9:**
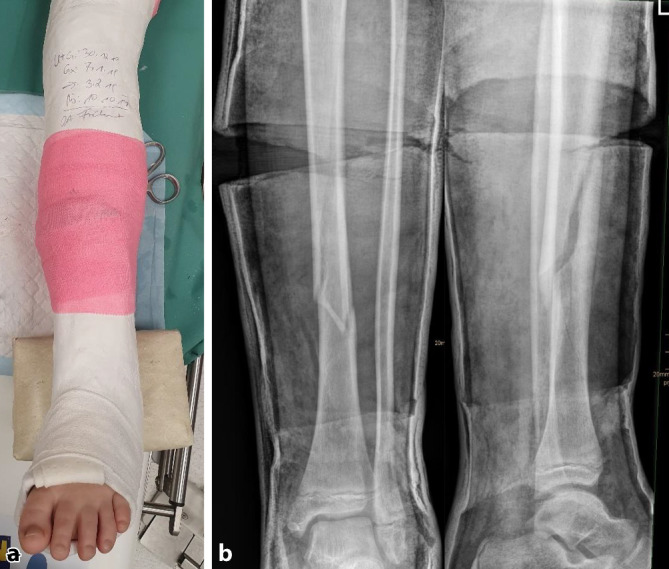
Als letzter Schritt wird die Keilungsstelle mit einer zirkulären Kunststoffbinde verschlossen, die im seltenen Fall einer notwendigen Nachkeilung, wobei der Spalt weiter aufgekeilt und ein größerer Platzhalter eingefügt wird, wieder entfernt werden kann (**a**). Das definitive Keilungsergebnis wird radiologisch dokumentiert (**b**)

### Technik am Unterarm

(Abb. 10, 11, 12, 13, 14, 15, 16, 17, 18, 19 und 20).

**Abb. 10:**
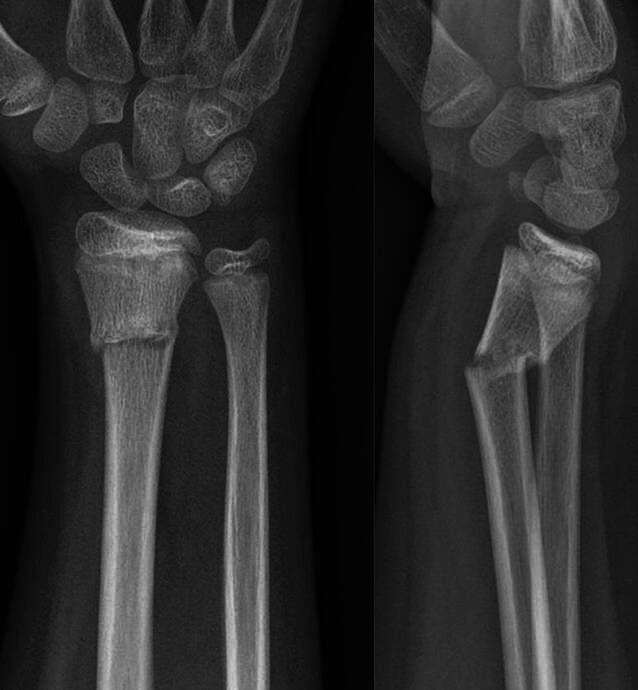
11-jähriger Junge, Sturz mit Skateboard. Distale metaphysäre vollständige Radiusfraktur mit Achsenknick nach dorsal von rund 30°. Am Unfalltag unter Analgetikagabe zur primären Schmerzbehandlung Anlage eines Oberarmspaltgipsverbandes in Vertikalextension mit gefühlvollem Anmodellieren entgegen der Fehlstellung

**Abb. 11:**
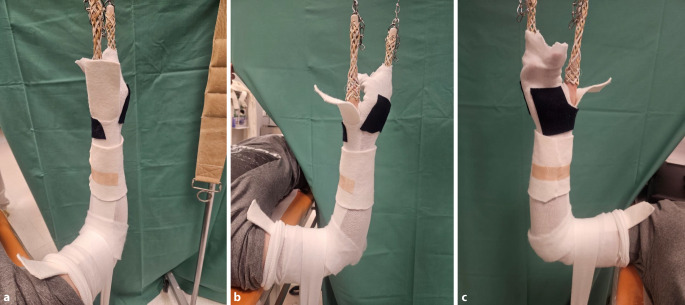
Am 5. Tag erfolgt *die Anlage des zum Keilen vorbereiteten Oberarmgipsverbandes.* Vor dem Klinikbesuch häusliche zeitgerechte Schmerzmittelgabe. In Rückenlage zunächst radialseitiges Anlegen des Filzstreifens (Schutz für die Haut bei der Gipsabnahme) und faltenfreies Anbringen des Strumpfes. Vertikalextension mit 2 „Mädchenfängern“ (= Finger-Strips/Extensionshülsen), der eigentliche Zug wirkt am Daumen, der fixierte Mittelfinger dient zur zusätzlichen Sicherung. Am Oberarm mithilfe eines Mullschlauches zusätzliche Stabilisierung durch Extension mit 1–2 kg Gewicht. Wattierung am Ellbogen und am proximalen Gipsende, Klebefilz zum Schutz vor Druckschäden am Handrücken sowie an der Handwurzel beugeseitig bis in die Hohlhand. Zirkuläres Anlegen des Filzstreifens im geplanten Keilungsareal. **a**–**c** Vorbereitung zur Anlage des zum Keilen vorgesehenen Gipsverbandes in Vertikalextension

**Abb. 12:**
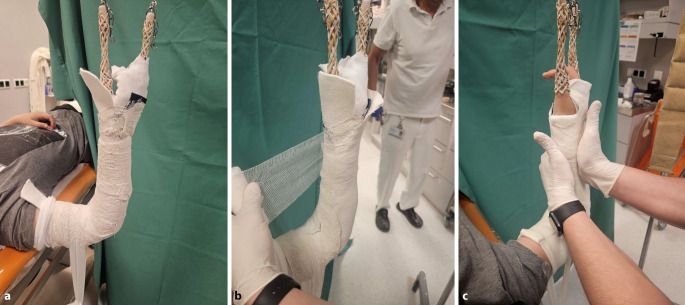
Dünnlagiges Anbringen einer zirkulären Gipsbinde (**a**), Auflegen einer Gipslonguette, die, beginnend an den Fingergrundgelenken streckseitig, steigbügelartig über das Ellbogengelenk bis zur proximalen Hohlhandbeugefalte gezogen wird. Die Streckung und Beugung der Langfinger müssen uneingeschränkt möglich sein. Zusätzliches Anlegen eines Gipsstreifens am Unterarm radial- und ulnarseitig, Fixierung der Longuetten am Handgelenk mit einer Mullbinde, die in Form von 3 Hohlhandtouren angebracht wird (**b**). Proximales und distales Umschlagen des Strumpfes und Vervollständigung des Oberarmgipses mit einer zirkulären Binde. Anmodellieren entgegen der Fehlstellung mit gestreckten Handflächen, wobei Falten unbedingt zu vermeiden sind (**c**)

**Abb. 13:**
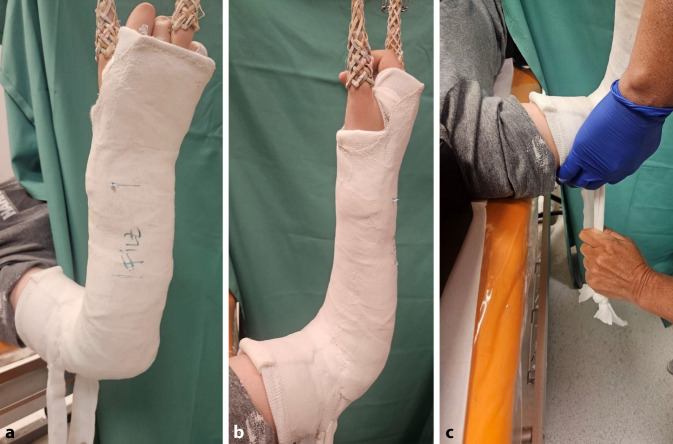
Zuletzt Einzeichnen des möglichen Keilungsareals (**a**, **b**) und Entfernung des Extensionsstrumpfes (**c**)

**Abb. 14:**
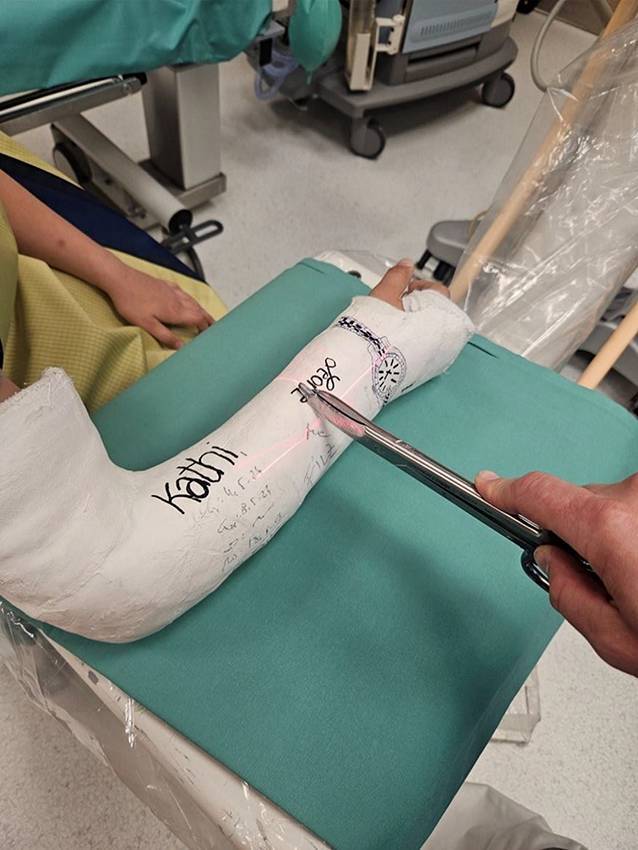
*Durchführung der Gipskeilung am Unterarm.* Am 8. Tag wird zur Beurteilung der aktuellen Dislokation eine Röntgenkontrolle angefertigt. Die Keilung wird im Regelfall im Sitzen durchgeführt, der Arm des Patienten ist auf der Platte des Bildwandlers gelagert. Unter Durchleuchtung erfolgt die Bestimmung der Keilungsstelle. Aufgrund der alleinigen Achsenabweichung zur Streckseite muss der Keil streng dorsalseitig gesetzt werden, um auf den konkaven Anteil der Fraktur zu wirken. Da der Bruch im distalen metaphysären Speichenabschnitt liegt, wird zur Gewährleistung einer ausreichenden Hebelwirkung die Höhe rund 2 bis 3 Querfinger proximal davon gewählt

**Abb. 15:**
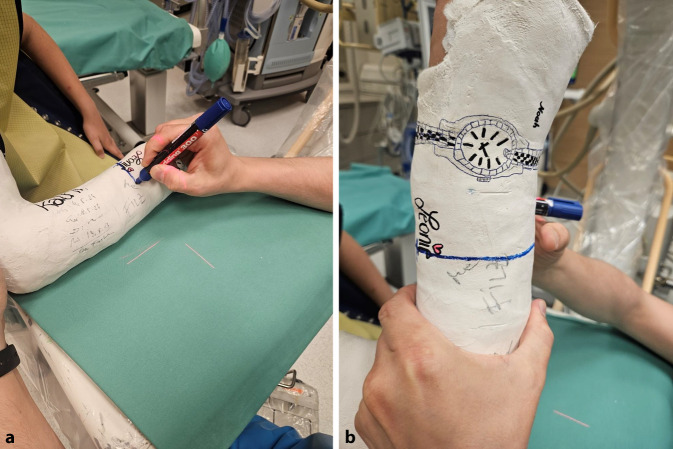
Anschließend erfolgt das Einzeichnen des Keilungsareals. Um die gewünschte Korrektur zu erzielen, muss die Linie 2–3 Querfinger proximal der Fraktur waagerecht auf die Unterarmachse verlaufen, um ein Aufkeilen zu ermöglichen, muss die Schnittführung semizirkulär angelegt sein. **a** Einzeichnung der Keilungshöhe, **b** definitive Keilungshöhe und Lokalisation

**Abb. 16:**
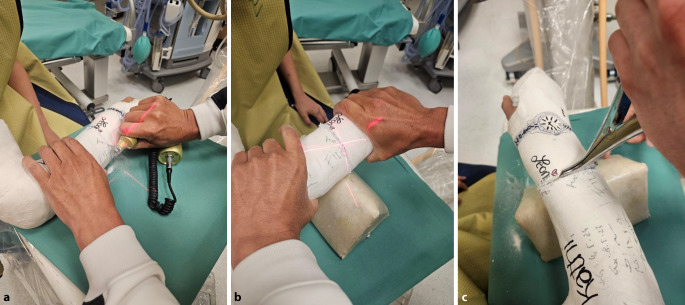
Einschneiden des Gipsverbandes entlang der Markierung, es ist darauf zu achten, dass alle Lagen des Gipses durchtrennt werden. Zur Stressverminderung für den Patienten wird die Verwendung einer geräuscharmen kleinen oszillierenden Säge mit Akkubetrieb empfohlen (**a**). Leichtes vorsichtiges manuelles Aufdehnen des Spaltes mithilfe des Keilpolsters (**b**) und mittiges Einsetzen des Spreizers (**c**)

**Abb. 17:**
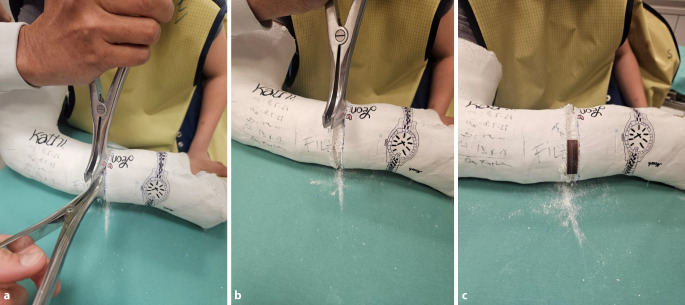
Dosiertes Aufkeilen (**a**) und Offenhalten des Spaltes mit einem zweiten, etwas randständig eingesetzten Spreizer (**b**). Einlegen eines passenden Keils (**c**)

**Abb. 18:**
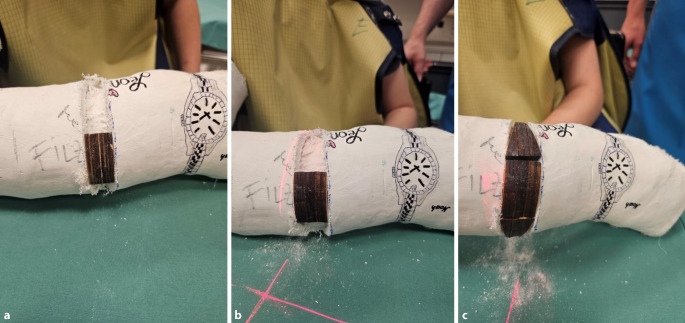
Schrittweises weiteres Aufdehnen und Einsetzen der entsprechenden Keile. Dieser Vorgang sollte langsam und unter entsprechender Kommunikation mit dem Patienten erfolgen. Äußert das Kind einen leichten ziehenden Schmerz im Bereich der Fraktur, ist meist das richtige Keilungsausmaß erreicht (**a**, **b**). Zur Vermeidung eines Fensterödems wird die Keilungsstelle vollständig mit Platzhaltern ausgefüllt, wobei an den beiden Enden ggf. auch zugeschnittene Korkstücke verwendet werden können. Um ein Nachlassen der Aufdehnung zu verhindern, sollte zumindest im Zentrum stabiles hölzernes Material zur Anwendung kommen (**c**)

**Abb. 19:**
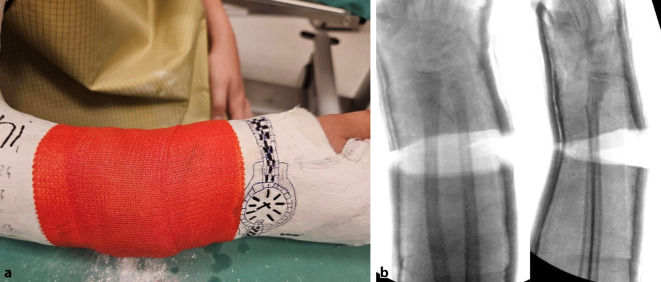
Es erfolgt der Verschluss des aufgekeilten Spaltes mit einer Kunststoffbinde (**a**), das Keilungsergebnis wird radiologisch dokumentiert. Es zeigt sich eine Restachsenabweichung zur Streckseite von knapp 8°. Da die definitive Wirkung der Keilung erst nach 3 bis 4 Tagen zum Tragen kommt und aufgrund der noch aktiven distalen Speichenfuge ein Restkorrekturpotenzial besteht, ist das Therapieziel erreicht (**b**). Der Patient verbleibt noch 20 min im Warteraum, dann erfolgt bei Beschwerdefreiheit und regulärer peripherer Durchblutung, Motorik und Sensibilität die Entlassung. Bei Schmerzen im Frakturbereich bzw. bei eingeschränkter Peripherie kann der Spalt durch Abnahme der Kunststoffbinde und Wechseln auf etwas kleinere Keile verschmälert werden. Drückt der Gipsverband außerhalb der Keilungszone, kann ein Gipsfenster zur Kontrolle und Entlastung der Haut angelegt werden. Sollten die Schmerzen nach beschriebenen Interventionen weiterbestehen, muss der Gips zur Gänze wieder entfernt werden. Selbiges Vorgehen gilt natürlich auch für die Keilung am Unterschenkel

**Abb. 20:**
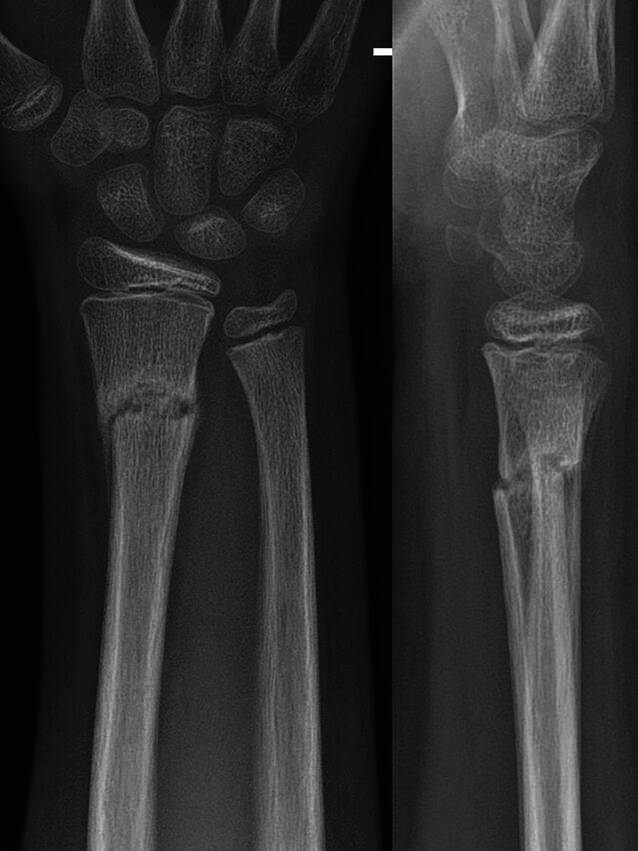
Belassen des gekeilten Gipsverbandes für insgesamt 4 Wochen ab Trauma, nach Gipsabnahme wird ein Konsolidierungsröntgen durchgeführt. Bedingt durch das weitere Redressement in den ersten Tagen nach der Keilung, kommt es zu einer weiteren Korrektur der Restachsenabweichung auf einen jetzt neutralen Speichenschaftgelenkwinkel in der Seitenaufnahme

**Abb. 21:**
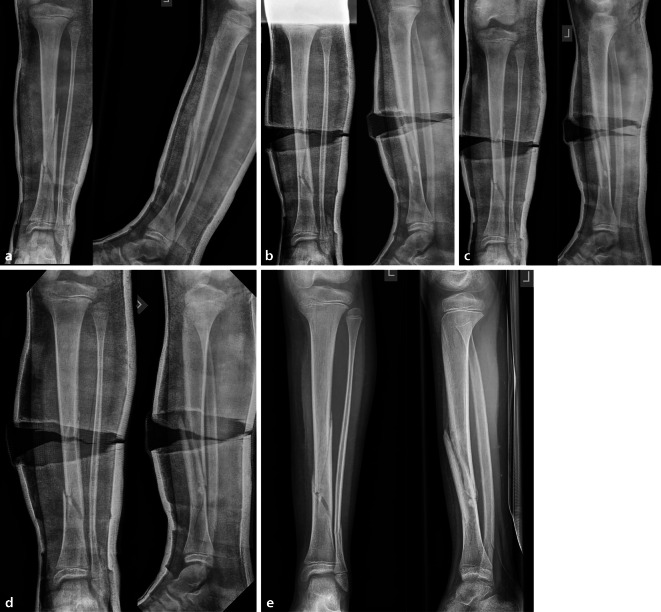
*Fallbeispiel Nachkeilung am Unterschenkel.* 9‑jähriger Junge, Tibiaspiralfraktur mit ausgebrochenem ventralen Biegungskeil im mittleren und distalen Schaftdrittel, Achsenabweichung in den Varus und in die Retrokurvation, zusätzlich Grünholzkomponente an der Fibula (**a**). Am 8. Tag erfolgt die Beseitigung der Fehlstellung durch anteromediale Gipskeilung (**b**). Aufgrund des langstreckigen komplexen Frakturverlaufes wird nach einer Woche eine Stellungskontrolle durchgeführt, die eine diskrete Redislokation in den Varus zeigt (**c**). Die Kunststoffbinde wird entfernt, die Korrektur erfolgt durch weiteres Aufdehnen des Keilungsspaltes und Einlegen eines größeren Platzhalters (**d**), das Konsolidierungsröntgen nach 5 Wochen zeigt eine achsengerechte Ausheilung (**e**)

## Allgemeine Voraussetzungen

Die konservative Behandlungsmethode mittels Gipskeilung von ausgewählten kindlichen Frakturen am Unterarm bzw. am Unterschenkel erfordert ein fundiertes Grundwissen über die Technik der korrekten Anlage eines Oberarm/Oberschenkelgipsverbandes sowie die dafür notwendige Infrastruktur (Gipsraum, Materialien, ausgebildetes Gipspersonal). Die Einhaltung eines vorgegebenen Zeitplanes vom Unfalltag bis zur Anlage des zum Keilen vorbereiteten Gipsverbandes (4.–5. Tag) und der eigentlichen Keilung (8.–10. Tag) ist zur Gewährleistung des Therapieerfolges unbedingt vonnöten. Voraussetzung hierfür ist, vor allem zu Beginn, ein gewisses persönliches Engagement der Behandler.

### Röntgenkontrollen

Standardmäßig erfolgt bei der Behandlung mittels Gipskeilung eine Röntgenaufnahme in 2 Ebenen zur Primärdiagnostik, vor und nach der Keilung sowie bei der Gipsabnahme zur Dokumentation des Ausheilungsergebnisses. In Ausnahmefällen, bei Frakturen am Unterschenkel, nochmalige radiologische Kontrolle eine Woche nach Keilung, um ggf. einen Stellungsverlust durch eine Nachkeilung ausgleichen zu können.

### Weitere Behandlung nach erfolgter Keilung

#### Unterarm

- Gipsbefristung für insgesamt 4 Wochen
- Konsolidierungsröntgen bei Gipsabnahme
- Sportfreigabe bei Schmerzfreiheit und uneingeschränkter Beweglichkeit
- Bei belassenen Fehlstellungen im altersabhängig tolerablen Bereich klinische Langzeitkontrollen bis zur achsengerechten Ausheilung

#### Unterschenkel

- Gipsbefristung altersabhängig für insgesamt 4 bis maximal 6 Wochen
- Bei älteren Patienten Teilbelastung mit Gehstöcken unter Abrollen des Beines
- Bei jüngeren Patienten Rollstuhl mit Beinstütze, ggf. „Selbstmobilisierung“ gegen Ende der Gipsbefristung
- Konsolidierungsröntgen bei Gipsabnahme
- Sportfreigabe bei Schmerzfreiheit, uneingeschränkter Beweglichkeit und normalem Gangbild, generell 8 Wochen nach Gipsabnahme
- Zur Kontrolle der Beinlänge Langzeitkontrolle über 2 Jahre in halbjährigen Intervallen

## Fehler, Gefahren, Komplikationen

**Abb. 22:**
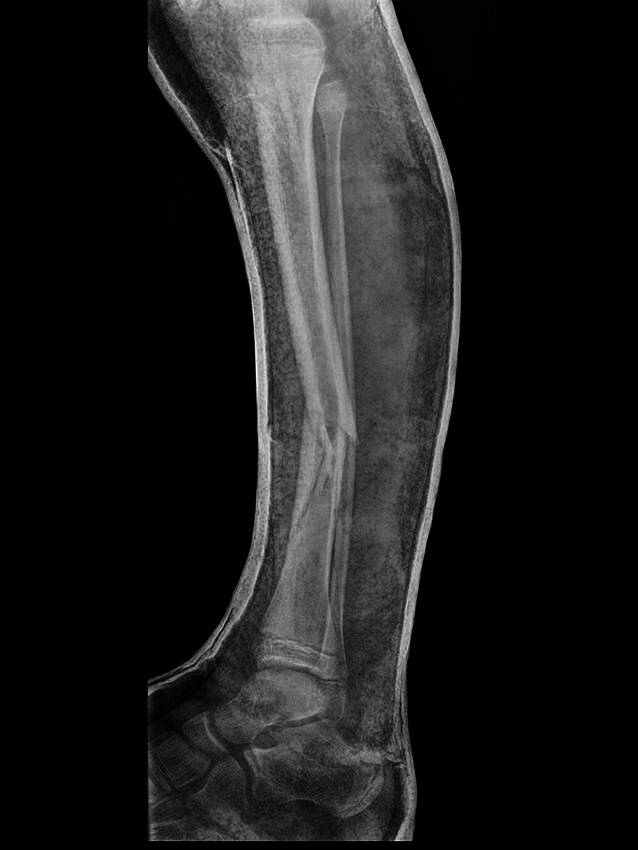
*Fehlerhafte Gipsanlage.* Durch alleiniges Halten des Beines am Sprunggelenk Provokation einer intolerablen Retrokurvationsfehlstellung

**Abb. 23:**
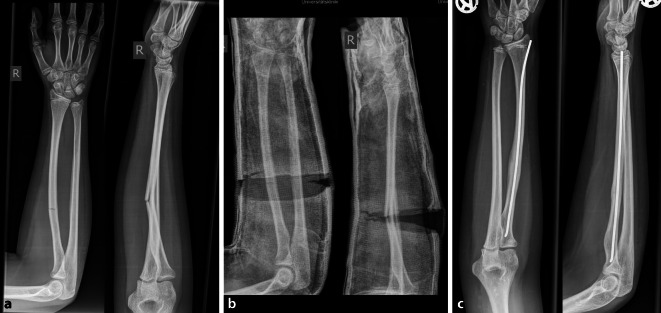
*Nichterreichen des Therapieziels.* 12-jähriges Mädchen, Grünholzfraktur Übergang proximales in mittleres Speichendrittel. Nichttolerable Achsenabweichung, kein Korrekturpotenzial, bei Belassen der Fehlstellung ist eine permanente Einschränkung der Vorderarmdrehung zu erwarten (**a**). Am 9. Tag frustraner Versuch der Stellungskorrektur durch Gipskeilung (ungeeignete Frakturlokalisation – muskulärer Weichteilmantel, unzureichende Hebelwirkung durch benachbartes Ellbogengelenk) (**b**). Verfahrenswechsel auf ESIN (elastische stabile intramedulläre Nagelosteosynthese). Das Ausheilungsergebnis zeigt eine achsengerechte Konsolidierung (**c**), klinisch ist der Patient beschwerdefrei bei uneingeschränkter Beweglichkeit

- Die Durchführung der Keilung in einem Anästhesieverfahren (Lachgas‑, Regional- oder Allgemeinanästhesie) wird in einigen Publikationen [9] vorgeschlagen. Nach Ansicht der Autoren ist dies aufgrund der geringen Schmerzbelastung nicht erforderlich und verhindert auch die Kommunikation mit dem Patienten, die für eine erfolgreiche Keilung ohne Komplikationen (v. a. Schmerzen durch nicht passende Gipsanordnung) vonnöten ist
- Verschlechterung der Frakturstellung durch fehlerhafte Gipsanlage (Abb. 22)
- Idealer Zeitpunkt der Gipskeilung ist der 8. bis 10. Tag nach Trauma, bei vorzeitiger Korrektur der Fehlstellung wird diese aufgrund der Schwellung und des noch nicht ausgebildeten Fixationskallus vom Patienten nicht toleriert, auch besteht die Gefahr der Provokation eines Kompartmentsyndroms. Bei Keilung zu einem späteren Zeitpunkt ist das Therapieziel wegen der fortgeschrittenen Knochenbruchheilung nicht mehr zu realisieren
- Persistierende Schmerzen im Frakturbereich, eingeschränkte periphere Durchblutung, Motorik und Sensibilität nach durchgeführter Keilung. Meist bedingt durch zu forciertes Aufkeilen, normalerweise kommt es zu einer Remission der Beschwerden durch Verkleinerung des Spaltes durch einen 2 mm kleineren Keil. Sollte noch eine Restkorrektur erforderlich sein, kann diese nach einigen Tagen bei Beschwerdefreiheit durch eine Nachkeilung erzielt werden
- Druckschmerzen nach Keilung außerhalb des Frakturbereiches, v. a. an Handgelenk, Handrücken, Fußrücken und Ferse. Kann durch Anlage eines Gipsfensters korrigiert werden, zur Vermeidung eines Fensterödems ist darauf zu achten, dass dieser Bereich zur Gänze durch einen Gipsdeckel wieder verschlossen wird
- Überkorrektur der Fraktur durch zu forciertes Aufkeilen. Prinzipiell sollte das Keilungsziel gerade erreicht werden, weil in den Folgetagen nach Keilung noch eine gewisse Restkorrektur stattfindet
- Sensibilitätsstörungen nach erfolgter Keilung (meist Medianussymptomatik bei volar verkippter Speichenfraktur durch beugeseitiges Aufkeilen). Remission durch Verkleinerung des Keiles, bei Persistenz der Beschwerden muss der Gipsverband entfernt werden
- Fehlkeilung durch falsch gewählte Keilungsstelle
- Stellungsverlust bei Tibiafrakturen mit Redislokation in den Varus (± Retrokurvation). Bei längerstreckigen Spiralfrakturen an der Tibia radiologische Stellungskontrolle auch 14 Tage nach Trauma und ggf. nochmalige Achsenkorrektur durch Nachkeilung (Abb. 21)
- Nichterreichen des Therapieziels, mangelhafte Korrektur der Fehlstellung (Abb. 23)
- Gefahr der Refraktur bei Grünholzfrakturen (v. a. am Unterarm) durch mangelnde Kompression und daraus entstehender Bruchheilungsstörung an der durchgebrochenen Konvexseite der Fraktur, weil die zur sicheren Heilung erforderlichen Hebelkräfte mitunter vom Patienten schmerzbedingt nicht toleriert werden

## Ergebnisse

Im Beobachtungszeitraum 2016 bis 2023 wurden an unserer Klinik 199 Gipskeilungen durchgeführt, 132 an der oberen und 67 an der unteren Extremität. Die Altersverteilung lag zwischen 15 Monaten und 16 Jahren, das Durchschnittsalter war 8,9 Jahre; 71 Patienten waren weiblich, 128 männlich.

An der *oberen Extremität* kam es in 4 Fällen zu einer Refraktur, wobei es sich hierbei immer um typische Grünholzfrakturen (Biegungsbrüche mit einseitiger Kortikalisunterbrechung bei sonst erhaltenem Periost) handelte. Insgesamt wurden 78 Patienten mit dieser Bruchform am distalen Unterarm mittels Gipskeilung behandelt, was einer Refrakturquote von rund 5 % entspricht, die damit deutlich unter den Literaturangaben liegt [8]. Bei einer Patientin wurde das Therapieziel mit der Gipskeilung nicht erreicht (falsche Indikationsstellung, Abb. 23).

In einigen wenigen Fällen, sowohl an der oberen als auch an der unteren Extremität, kam es zu minimalen Hautirritationen, die nach Gipsabnahme in kurzer Zeit problemlos abgeheilt sind.

An der *unteren Extremität* war bei einer Patientin mit vollständiger distaler metaphysärer Tibiafraktur und intolerabler Varusfehlstellung nach frustraner Keilung ein Verfahrenswechsel auf eine geschlossene Reposition und gekreuzte Kirschner-Draht-Osteosynthese erforderlich. In einem weiteren Fall konnte die Keilung aufgrund von insuffizienter Gipsanlage nicht durchgeführt werden (Abb. 22). Bei einem 15 Monate alten Knaben mit proximaler metaphysärer Biegungsfraktur an der Tibia musste aufgrund schlechter klinischer Beurteilbarkeit der gekeilte Gipsverband nach einigen Tagen gewechselt werden.

Bei den restlichen Patienten (96 %) konnte das Therapieziel durch die Gipskeilung erreicht werden, vergleichbare Ergebnisse sind auch in der Literatur zu finden [3, 5].

Detaillierte klinische Auswertungen (z. B. Wiedererlangen der freien Funktion, Wiederaufnahme von Sport, visuelle Analogskala bezüglich der Schmerzwahrnehmung vor, während und nach der Keilung) und biomechanische Analysen werden derzeit in einer gemeinsamen Studie der Kliniken in Bern und St. Pölten ausgearbeitet. Hier zeigen sich in der präliminären Auswertung erfreuliche Ergebnisse, die in naher Zukunft veröffentlicht werden.

## Data Availability

Die in dieser Studie erhobenen Datensätze können auf begründete Anfrage beim Korrespondenzautor angefordert werden.
